# Using a surrogate human digital twin to model embodied perception and cognition for neuroergonomics

**DOI:** 10.3389/fnrgo.2026.1939412

**Published:** 2026-09-21

**Authors:** Tajbeed A. Chowdhury, Alexander L. Francis, Daniel J. Strauss

**Affiliations:** 1 Systems Neuroscience and Neurotechnology Unit, Faculty of Medicine, Saarland University & School of Engineering, htw saar, Saarbrücken, Germany; 2 Center for Digital Neurotechnologies Saar, Saarland University & htw saar, Saarbrücken and Homburg/Saar, Germany; 3 Department of Speech, Language, and Hearing Sciences, Purdue University, West Lafayette, IN, United States

**Keywords:** active inference, attention mechanism, cognitive modeling, free energy principle, human digital twin, neuroergonomics, sensory conflicts, surrogate digital twin

## Abstract

Understanding and predicting human behavior in dynamic, multisensory environments is one of the central challenges in neuroergonomics and human-centered artificial intelligence. Computational neuroscience provides mechanistic theories of perception, attention, and decision-making, but these insights have not yet been fully integrated into real-time digital representations of human physical behavior. Conversely, Human Digital Twins (HDTs) can successfully represent human physical characteristics, especially biomechanics and physiology, but remain limited in capturing the added complexities of embodied perception and cognition. Here, we introduce the concept of a Surrogate Human Digital Twin (S-HDT), emphasizing the necessarily approximate nature of the proposed surrogate model. Drawing upon active inference theory and consideration of evolutionary priors, the S-HDT operates as a generative model, continuously computing hidden states of cognition within a human model and predicting their trajectories under a changing environment. We argue that a S-HDT can serve as a computational approximation of embodied human perception and cognition as they function in modern multisensory environments. We present a proof-of-concept S-HDT simulating aspects of human attention and motivation mechanisms within complex sensory environments. The simulation illustrates the behavior of selected components of our proposed framework rather than providing empirical validation of human cognitive-state prediction. Future work should focus on multimodal human-data validation, and application-specific operationalization. This framework opens new opportunities for investigating neuroergonomic system design, human-centered artificial intelligence, and adaptive human-machine interaction.

## 1 Introduction

The concept of the Human Digital Twin (HDT) has surfaced as a strong computational paradigm for mimicking human characteristics in virtual environments (Lin et al., 2024; Sparrow et al., 2019; Chowdhury et al., 2026a). However, existing approaches primarily concentrate on physical characteristics, especially musculoskeletal models, physiological monitoring systems, and biomechanical simulations (Asad et al., 2023). These HDTs are often designed to predict body movement, muscle fatigue, workload, or injury risk in certain environments, as shown in our previous work (Chowdhury et al., 2024a,b). However, contemporary environments increasingly challenge human cognition rather than or in addition to human physiology (Pan et al., 2025). Modern humans typically operate within complex multisensory environments characterized by multiple, competing sensory streams leading to information overload, sensory conflict and sensory strain (Strauss et al., 2025; Corona-Strauss et al., 2026; Luo et al., 2024; Hoover, 2021). Operating in such environments places significant demands on the perceptual and attentional resources of humans and generates a high level of uncertainty. Thus the effects of such environments on human behavior cannot be accounted for by modeling physical characteristics alone (Lazaros et al., 2026).

Despite significant advances in human-centered sensing, cognitive modeling, and computational neuroscience, current neuroergonomic systems remain largely descriptive rather than predictive (Diarra et al., 2025). Many existing approaches focus on *post hoc* analysis of physiological signals or behavioral markers to infer human cognitive states (Reid and Nygren, 1988; Jin et al., 2025). While these methods have enabled important progress in understanding human performance in complex environments, they are fundamentally limited in their ability to anticipate future cognitive states or support proactive adaptation (Jin et al., 2025). These frameworks do not have the ability to capture the ongoing engagement of cognition as a continuous and dynamic temporal process. As a result, these models fail to generalize across tasks, individuals, and different real-world conditions (Chowdhury et al., 2026a). Toward that end, recurrent architectures such as Long Short-Term Memory (LSTM) can model dependencies across extended temporal sequences (Hochreiter and Schmidhuber, 1997), while transformer-based architectures can also enable long-range dependencies and forecasting (Wen et al., 2022). Additionally, reinforcement learning approaches further enable sequential decision-making and model adaptation (Zhang R. et al., 2022). However, modeling with transformer-based architectures relies on a passive, data-driven approach (Parr et al., 2025). Similarly, with reinforcement learning, the modeling paradigm is dependent on reward engineering and brute force, where trial-and-error exploration is the primary driving theme (Tschantz et al., 2020). However, biological attention is defined as precision weighting, which the other approaches do not account for (Brown et al., 2011). Additionally, in the existing work on cognitive HDTs, there remains a significant challenge in integrating heterogeneous and multimodal human observations into an interpretable and continuously updating embodied artificial intelligence (Lin et al., 2024; Zhong et al., 2023). Physiological signals, behavioral data, and environmental metadata are often treated independently (Chowdhury et al., 2025). Some existing implementations rely on a limited number of data modalities or model a particular human state separately (Chowdhury et al., 2026b; Zhong et al., 2023). This leads to an incomplete representation of the current human state. Recent research in the field of neuroergonomics further emphasizes overlapping physiological signals across cognitive states and argues for the need for a standardized framework with multimodal representations and not conditions (workload, stress, etc.) in isolation (Rivas-Vidal et al., 2025). On top of that, uncertainty-aware inference, bidirectional perception-action coupling, and counterfactual simulation are yet to be integrated within a common generative modeling of HDT implementation (Murray-Smith et al., 2025). Thus, there is a need for a unified computational framework to link models of perception, cognition, and physical behavior in order to more fully represent the complex, bi-directional interaction between humans and their surrounding environment (Clark, 2013; Berti et al., 2024). This is especially the case in closed-loop settings where human cognitive states continuously influence and are also influenced by system behavior (Zhang N. et al., 2022).

These limitations create the need for a new class of modeling paradigms in neuroergonomics that move beyond passive inference toward predictive and generative representations of human cognition. In particular, there is a growing need for systems that can simulate latent cognitive states, anticipate cognitive transitions, and support adaptive interventions in real time. We therefore introduce the concept of a Surrogate Human Digital Twin (S-HDT). By integrating multimodal sensing with generative computational models, S-HDTs aim to provide a continuously updating surrogate of embodied human perceptual and cognitive state dynamics. Grounded in active inference (Friston et al., 2006), this framework has the potential to unify perception, learning, inference, and prediction across the nested temporal horizons and spatial scales of the embodied agent's environment. We have previously termed this structure, metaphorically, a *spatiotemporal scale space* (Strauss et al., 2025). While traditional HDTs attempt to replicate observable aspects of a human system (Bruynseels et al., 2018), S-HDTs are not attempting to imitate the human directly. In HDTs, the objective is often to establish a digital counterpart that mirrors the state of the physical individual. In contrast, S-HDTs model the latent computational processes that are presumed to generate human embodied perception and cognition. Rather than replacing the existing approaches of modeling, S-HDT aims to address a complementary modeling objective that maintains an explicit probabilistic generative model connecting latent cognitive states, environmental context, and action within a continuously updating action-perception loop. We consider active inference for the proposed S-HDT because it provides a formal framework for belief updating under uncertainty. We do not assume that active inference provides a superior predictive outcome to recurrent, transformer-based, or reinforcement learning approaches, which remains an empirical question requiring comparative evaluation. While a conventional HDT asks the question of,

“*What observable physical, physiological, and behavioral state is the person currently in?”*

The proposed S-HDT aims to address a complementary level of description. Based on the important evidence from observable physiological states, S-HDT extends to answer,

“*What does the human currently believe about the world, what are they attending to, and how are they minimizing uncertainty?”*

The S-HDT therefore, operates as a probabilistic model (including of inferred, hidden cognitive states) rather than a deterministic representation of purely observable physical variables. Ultimately, this shift from reactive measurement to predictive cognitive modeling opens the door to a new paradigm in neuroergonomics (Ayaz, 2025; Barresi, 2024), in which human cognitive states are not only imputed from physical measures *post hoc* but also anticipated and potentially shaped to enhance performance, safety, and wellbeing in complex real-world environments.

## 2 Conceptual model of S-HDT

The S-HDT is designed as a computational abstraction of human cognitive dynamics, enabling predictive, adaptive, and personalized modeling of neuroergonomic states. Unlike conventional human modeling approaches that rely on static feature representations or short-term statistical inference, S-HDT is grounded in a generative and inferential framework that continuously updates its internal belief about the operator's latent cognitive state based on multimodal observations. Figure 1 presents the conceptual architecture of the proposed S-HDT. The left-hand side of Figure 1 represents the multimodal sensory interface through which S-HDT observes the human operator and the surrounding environment. Human cognition is inherently multimodal, integrating information from both external and internal sensory channels (Barrett, 2017; Strauss and Francis, 2017; Strauss et al., 2024). Accordingly, the framework considers an example set of three core sensory streams shown in blue and two physiological and inferred sensory inputs shown in magenta. The type of multisensory input can be expanded or reduced depending on the application and model objective.

**Figure 1:**
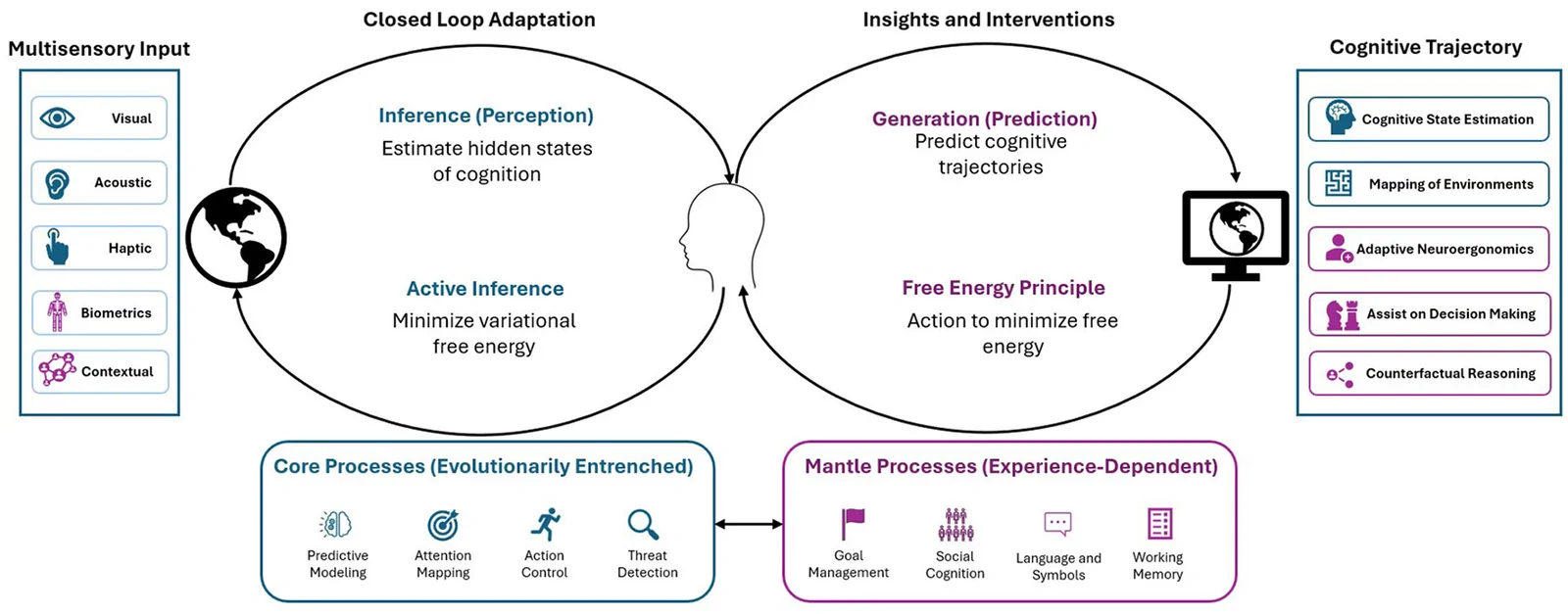
A conceptual framework of the proposed S-HDT operating in a closed loop scenario.

Together, these heterogeneous sensory streams form the observable evidence used by the generative model to infer hidden cognitive states. The framework is inspired by the active inference paradigm, where cognition emerges from a continuous interaction between perception, prediction, and action (Pezzulo et al., 2024).

### 2.1 Active inference inspired computational foundation for S-HDT

The fundamental computational mechanism of our S-HDT model is inspired by the active inference principle. The model considers cognition, perception, and subsequent action as not separate components but part of one unified process of probabilistic inference (Murray-Smith et al., 2025; Pezzulo et al., 2024). After sensory acquisition, multimodal observations enter the active inference engine as input. Within the multimodal observations, each channel contributes evidence with an associated degree of reliability or precision. When observations are mutually consistent, different modalities can jointly constrain the inferred state. And during conflict, the model may assign greater influence toward the more reliable or contextually relevant modalitiy while retaining uncertainty regarding alternative explanations. In this framework, the system maintains a generative model of the world that includes representations of hidden causes derived from input data. These causes may include cognitive load, attention, fatigue, emotion, and task engagement, to name a few, and they are derived from input streams obtained from the operator and environment including for example physiological measures. The S-HDT continuously updates its beliefs about these hidden states by minimizing the discrepancy between predicted and observed sensory data. This distinctness minimization results in progressively more accurate representations of the operator's current cognitive state. Consequently, cognition is characterized not as a fixed label but as a continuously evolving probability distribution over latent mental states. In a neuroergonomic context, active inference enables the S-HDT to move beyond passive state estimation toward predictive modeling of cognitive trajectories. This allows the S-HDT to support anticipatory adaptation in human–machine systems, where interventions can be triggered before performance degradation occurs. Furthermore, active inference naturally supports multimodal integration, making it well-suited for neuroergonomic applications where physiological signals (e.g., heart rate variability, muscular activity), behavioral cues, and environmental context must be combined into a coherent latent representation of operator state (Thinnes et al., 2026). As a perspective, the S-HDT defines the conceptual and computational requirements rather than suggesting a universally applicable generative model implementation. The specific relation dynamics of elements are expected to depend on the task, sensing modalities and must therefore be instantiated and empirically validated in application specific implementations.

### 2.2 Free energy minimization

At the very center of active inference is the idea that systems can work toward minimizing free energy within Anthropocene sensory environments (Habib et al., 2007; Slabbekoorn, 2018), an approach that allows for practical goals for inference and learning within the bounds of S-HDT. The variational free energy acts as a ceiling bound on surprise elements such as mismatches between the auditory system's evolved expectations and contemporary soundscapes (Francis and Strauss, 2026). Thus, the system is capable of approximating Bayesian inference over hidden cognitive states without requiring explicit computation of intractable posterior distributions. The idea builds on the work of (Strauss et al., 2025), who applied the free-energy minimization principle to model effortful listening. The formal expression of variational free energy can be expressed as,

$$\begin{array}{l} F\left(Q,o\right)=E_{Q\left(s\right)}\left[-\ln\;p\left(s,o\right)\right]-H\left[Q\left(s\right)\right]\geq -\ln\;p\left(o\right). \end{array}$$ (1)

In the context of this paper, *Q*(*s*) is the surrogate twin's approximate posterior over hidden cognitive states *s*, *o* are the observations available to the twin, and *H*[*Q*(*s*)] is the entropy of the inferred belief distribution. Here, *Q*(*s*) asks the question,

“*What do I currently believe?”*

The expectation term *E*_*Q*(*s*)_[−ln *p*(*s, o*)] reflects how well the internal generative model of the surrogate twin explains the observed data in light of its current belief. So this expectation term asks the question,

“*How well do my beliefs explain what I observe?”*

In other words, this part measures the prediction error of S-HDT. The entropy term *H*[*Q*(*s*)] reflects the uncertainty about these beliefs. In this part, a high entropy means several explanations are still possible, whereas a low entropy suggests that the model is very confident. As in the formulation proposed in active inference, the inequality shows that variational free energy is an upper bound on surprise, which means that minimizing free energy is a way of minimizing the unexpectedness of incoming observations. However, free-energy minimization should not be understood as minimizing environmental complexity itself. Surprise is relative to the predictions and expectations of the generative model and to the operator's current goals. In complex multisensory environments, such as a cocktail party, the operator actively selects and samples task-relevant information through attentional allocation, gaze, head orientation, and other actions. Thus, the S-HDT keeps a suitable level of uncertainty for adaptive inference by always updating its internal belief distribution *Q*(*s*) to make its generative model more consistent with observations as shown in Equation [1].

### 2.3 Core and mantle cognitive processes

The lower portion of Figure 1 distinguishes between two complementary categories of cognitive function (Ribic, 2020), which we refer to as core and mantle processes. This distinction is intended as a functional modeling abstraction rather than a strict anatomical or evolutionary dichotomy. The sole purpose of the distinction is to provide an organizational structure for representing cognitive processes within S-HDT. The distinction allows the surrogate model to organize processes with different temporal and contextual characteristics while retaining their continuous and bidirectional interactions. The core processes correspond to evolutionarily conserved mechanisms that operate across most vertebrate species (Corona-Strauss et al., 2026). These include predictive modeling of sensory information, attentional allocation, action control, and threat detection (Heyes, 2018; Thinnes et al., 2025). They provide the fundamental computational infrastructure that enables organisms to survive in uncertain environments by rapidly predicting and responding to external events (Parr et al., 2022). Whereas mantle processes represents higher-order cognitive functions that are strongly shaped by learning, culture, and experience (Chiao and Ambady, 2007; Han and Northoff, 2008). These include goal management, social cognition, language processing, and symbolic reasoning. These functions enable humans to perform complex planning, abstract reasoning, communication, and collaborative decision making. The S-HDT integrates both categories of cognition within a unified generative framework. Core processes govern rapid perception-action loops, whereas mantle processes support long-term planning and adaptive reasoning (Daw et al., 2005).

### 2.4 Predictive cognitive simulation

One of the proposed capability of the S-HDT framework is the ability for predictive cognitive simulation, in which a sufficiently validated implementation could go beyond the real-time estimation of current states and to generate plausible future trajectories of human cognitive states. This capability is important in neuroergonomic environments for predicting performance degradation, workload surges, or attentional lapses before they become apparent in observable behavior (Hancock and Matthews, 2019). As more observations become available, the internal cognitive representation becomes increasingly personalized (Li et al., 2025). First, the framework performs cognitive state estimation, continuously monitoring latent mental states, biometric and contextual data when available throughout task execution. Second, it constructs environmental mappings that relate specific task contexts, environmental conditions, and interaction patterns to cognitive states. These two are the core processes of an S-HDT, shown in blue on the right side of Figure 1. Third, these models support adaptive neuroergonomics, where interfaces, task allocation strategies, or assistance levels are personalized according to the predicted cognitive needs of individual users. Fourth, S-HDT functions as an intelligent decision-support system, assisting operators and supervisors by recommending interventions that minimize cognitive overload while maintaining productivity and safety. Finally, the generative nature of the model enables counterfactual reasoning, allowing the system to answer what-if scenarios (Van Hoeck et al., 2015). In this perspective, we propose counterfactual reasoning as a conceptual capability rather than a fully specified computational module. In the context of active inference, this can be achieved by changing either the prior beliefs or the sensory inputs within the generative model and then propagating their effects forward through the inference process (Da Costa et al., 2020). The effects of changes in task difficulty, interaction velocity, or environmental stressors on physiological metrics such as heart rate variability or muscle activity can be evaluated (Borghini et al., 2014). These simulated scenarios enable the evaluation of alternative operational strategies without exposing human operators to unnecessary risk.

## 3 Simulation results: predictive cognitive dynamics under environmental complexity

As an example of the proposed S-HDT behavior, we present a simulated comparison between two representative neuroergonomic scenarios. One scenario is a quiet environment with low external distraction, and the other is a cocktail party environment, which is characterized by multiple competing sensory stimuli and increased environmental uncertainty (Yost, 1997; Bregman, 1994). The simulation deliberately instantiates only a restricted subset of the broader S-HDT framework, namely auditory attention and associated perception–action dynamics. It does not operationalize the complete multimodal or core–mantle architecture introduced in Figure 1. Furthermore, we intentionally simplify the cocktail-party condition without modeling speech intelligibility, linguistic content, informational masking, semantic relevance, or audio–linguistic interactions. The simulation is developed as a parameterized proof-of-concept environment for illustrating auditory-attention and prediction-error dynamics under different levels of sensory competition. It is not intended to constitute an acoustically validated reproduction of a real cocktail-party environment. It is composed of two synthetic environments, a quiet environment and a cocktail party environment, both of which generate structured auditory feature vectors from parametrized sound events, such as speech, laughter, glass clinks, footsteps, and competing speakers. The auditory scenes are modeled by 64-dimensional binaural spectral feature representations. The spatial information (azimuth, distance, and S-HDT head orientation) is modeled by left-right gain modulation and frequency-dependent spectral shaping (Blauert, 1997). In the cocktail party condition, event scheduling introduces temporal dynamics, yielding non-stationary and temporally evolving distractor streams. The sensory inputs are processed through an illustrative hierarchical scale-space predictive processing model, which calculates multi-level prediction errors ranging from spectral features to abstractions at the semantic level through increasingly abstract representations (Hohwy, 2013). The model adopts belief updating as a free-energy minimization principle that allows adaptive inference in uncertainty. The robot agent interacts with the environment in a closed-loop fashion by continuously updating its heading and attentional focus based on prediction error dynamics. In addition, abrupt free energy increases induce exogenous attentional shifts, simulating reflexive redistribution of cognitive resources under high cognitive load. These simulations do not provide an empirical validation of the active inference-based framework of cognitive dynamics but rather show its predictive power at different levels of sensory complexity. The resulting free energy dynamics, attentional behavior, tracking performance, and cognitive effort across conditions are summarized in Figure 2. All of these quantities are consistently larger in the cocktail party scenario than in the quiet environment. The results suggest that complex and rich stimulus environments involve greater levels of uncertainty and prediction errors, requiring the operator to continually update beliefs to maintain an accurate representation of the latent cognitive state. Collectively, these findings demonstrate the ability of S-HDT to perform continuous predictive cognitive monitoring, providing a foundation for proactive neuroergonomic interventions before observable performance degradation occurs.

**Figure 2:**
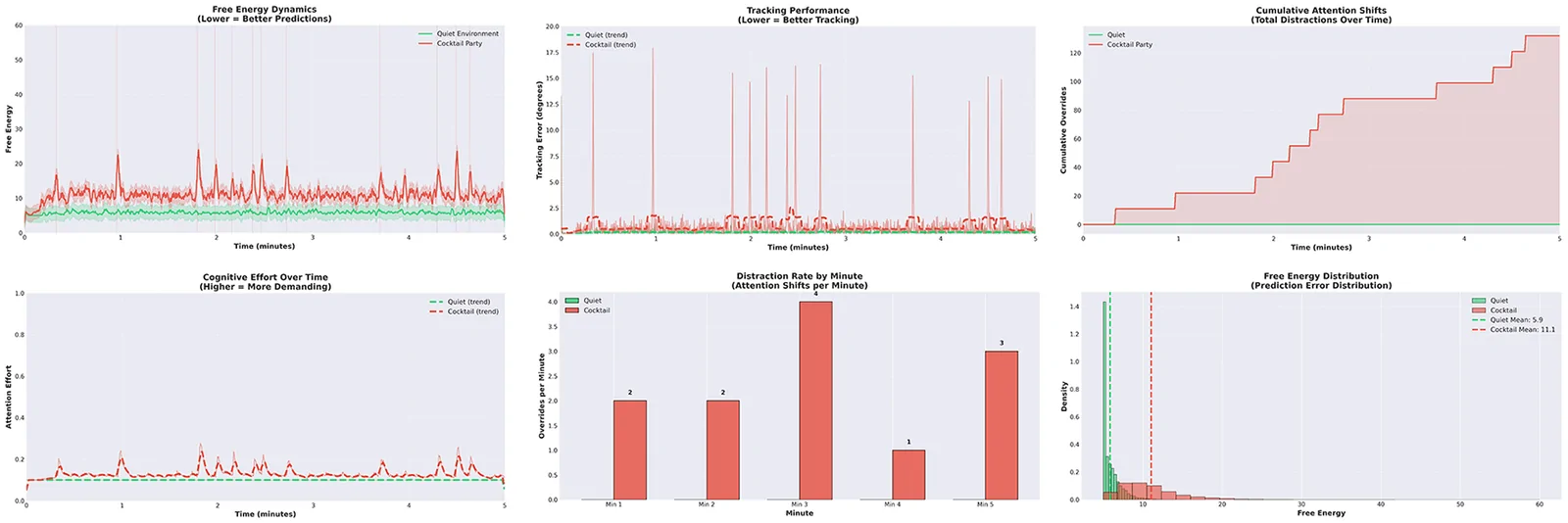
Evaluation of the 5-min simulation comparing quiet and cocktail party environments. The dashboard aggregates key behavioral and inferential metrics, including free energy, tracking accuracy, attentional effort, and exogenous override frequency.

## 4 Discussion and implications for personalized neuroergonomics

From a neuroergonomic perspective, this personalization concept of S-HDT is particularly critical in real-world environments where cognitive demand is highly context-dependent. Industrial assembly, teleoperation, and human–robot collaboration scenarios are characterized by rapidly changing task constraints, environmental uncertainty, and heterogeneous operator behavior. S-HDT addresses this variability by embedding prediction directly into the inference loop, allowing the system to anticipate cognitive transitions rather than merely classify current states. This enables proactive adaptation strategies that can mitigate performance degradation before it occurs. Another key implication lies in the unification of perception, cognition, and action under a single computational principle. Despite these advantages, several challenges remain in the operationalization of S-HDT for large-scale neuroergonomic deployment. First, the identifiability of latent cognitive states remains a fundamental limitation, as multiple internal states may produce similar observable physiological or behavioral patterns. The identifiability limitation deserves closer formal treatment than a Perspective allows. Within active inference, the relationship between the biosignals the S-HDT observes (e.g., cardiac, electrodermal, or muscular activity) and the latent affective, motivational, and attentional states it infers is typically framed through a Markov blanket: a statistical boundary that renders internal states conditionally independent of the environment given the sensory and active states of the blanket. This construct offers a principled vocabulary for the interface between human and environment, but its use as a warrant for reading internal states off physiology warrants caution. This is especially clear for the affective states the S-HDT aims to infer: interoceptive inference accounts treat emotion not as a hidden variable lying passively behind the blanket but as a construction that the generative model builds from interoceptive signals together with contextual and conceptual priors (Seth, 2013; Seth and Friston, 2016; Barrett, 2017), so that the same peripheral pattern can support different affective construals. Several structural limits follow. A Markov blanket is defined relative to a chosen model, level of coarse graining, and timescale, so the boundary is not independent of the observer; the mapping from peripheral signals to internal states is therefore many to one and not invertible (autonomic non-specificity, and, for affect, the absence of fixed physiological signatures); coupling between brain and body is dense and reciprocal rather than sparse, so the conditional independence holds only approximately; and because physiological variables are actively regulated (allostasis), they act simultaneously as evidence about and control over internal states, which makes the boundary partly circular. Accordingly, active inference should be read as constraining the state identifiability problem for the S-HDT, not dissolving it. Importantly, this limitation does not imply that probabilistic inference of latent states is without utility. Rather, it limits the extent to which an inferred state can be interpreted as a uniquely identifiable representation of the operator's internal cognition. Our proposed S-HDT therefore treats latent cognitive states as probabilistic surrogate representations whose uncertainty should be retained explicitly when multiple internal explanations remain compatible with the available observations. Clarifying when biosignals can be treated as a cleanly defined blanket around neuroergonomically relevant states, and quantifying the approximation error when they cannot, is an important direction for future work.

Active inference offers a principled probabilistic framework for representing and updating beliefs under this ambiguity, rather than resolving structural non-identifiability itself, but practical implementations still require careful model design and validation against ground truth proxies. Second, there are computational and synchronization limitations to real-time multimodal data fusion, especially when fusing high-frequency kinematic and physiological streams in simulation environments. Potential implementation strategies may include modality-specific pre-processing, asynchronous or multi-rate fusion, sliding temporal windows, dimensionality reduction, and event-triggered updating instead of processing every modality at its native sampling frequency. The computational feasibility and latency of these alternatives should be evaluated empirically for each deployment scenario. Another issue is generalization and transferability. Although S-HDT does stress personalization, it still needs a tradeoff between individual-specific modeling and cross-user generalization, especially when the amount of calibration data is limited. One possible solution is to use hierarchical or population-informed priors, which could help constrain individual models within larger neuroergonomic distributions. This direction is particularly important for scaling S-HDT from lab settings to industrial and clinical environments. Ethical and human factors considerations are also critical in deploying predictive neuroergonomic systems. The continuous inference of cognitive states raises issues of transparency, user autonomy, and data privacy. The S-HDT aims to improve performance and wellbeing, but its use to monitor cognitive load or fatigue must be carefully managed to avoid misuse or over-reliance on automated decision systems. Therefore, it is important to design the models with explainability of predictions and human-in-the-loop control. Concrete safeguards may include communicating prediction uncertainty to users, requiring human confirmation before consequential adaptive interventions. This will allow human-in-the-loop to pause or override monitoring and system actions. Overall, S-HDT is a step forward in the direction of a new class of simulation-driven neuroergonomic systems. This creates new avenues for improving human performance, safety and adaptability in complex socio-technical environments, but also raises significant challenges in terms of validation, scalability and ethical deployment.

## 5 Conclusion

This paper introduces the S-HDT as a conceptual computational framework for predictive and personalized neuroergonomics. By integrating multimodal sensing, generative modeling, and real-time simulation within a digital twin environment, S-HDT moves beyond traditional neuroergonomic approaches that are primarily descriptive or reactive. Instead, it establishes a closed-loop system in which human cognitive states are continuously inferred, simulated, and anticipated. At the core of S-HDT lies active inference, supported by the principle of free energy minimization, which provides a unified computational foundation for perception, learning, and decision making. This enables the system to maintain a continuously updating generative model of latent cognitive states such as workload, fatigue, and attention, inferred from heterogeneous data streams including kinematics, physiological signals, and behavioral cues. Importantly, this formulation allows S-HDT not only to estimate current cognitive states but also to predict their evolution under varying task and environmental conditions. However we do not establish the empirical accuracy, generalizability, or real-time effectiveness of these capabilities. The auditory simulation presented here provides an illustrative example of selected S-HDT principles rather than validation of the complete multimodal framework. The integration with a digital twin environment further enables real-time embodiment of these computational processes, bridging the gap between theoretical cognitive modeling and practical neuroergonomic applications. Through this coupling, S-HDT supports adaptive interaction strategies, including workload balancing, interface adaptation, and anticipatory assistance in complex human–machine systems. Overall, S-HDT represents a step toward a new paradigm in neuroergonomics, where cognitive modeling is no longer static or retrospective but becomes dynamic, predictive, and personalized. While challenges remain in terms of scalability, validation, and ethical deployment, the proposed framework provides a foundation for future research in intelligent, human-centered digital twin systems capable of enhancing performance, safety, and wellbeing in real-world environments.

## Data Availability

The raw data supporting the conclusions of this article will be made available by the authors, without undue reservation.
